# Habit and Illness
*A Paper read at a Meeting of the Bristol Medico-Chirurgical Society on Wednesday, 9th January, 1935.


**Published:** 1935

**Authors:** H. H. Carleton

**Affiliations:** Physician, Bristol General Hospital


### HABIT AND ILLNESS.*

BY

H. H. Carleton, M.D., M.R.C.P.,

*Physician, Bristol General Hospital.*

---

The symptoms of illness may appear as a medley of isolated phenomena, or they may be recognized as parts of some general reaction. In a doctor the capacity to appreciate an underlying unity amidst apparent diversity is a mark of efficiency. The man with the widest possible outlook usually makes the best doctor. Multiple diagnoses should be made with reluctance, and are more likely to be wrong than right.

There are two factors which interfere with breadth of view in clinical work: one is ignorance, which hardly requires discussion, the other is prejudice. Our outlook is liable to be hampered by our particular interests. How easy it is to persuade ourselves that we see what we want to see. Specialism in our profession is so necessary and useful that it requires no special pleading, the dangers of over-specialization are, however, not infrequently the products of a narrow outlook, slender knowledge, or even attenuated honesty. The definition of the word "cure" should always be suspect. I find it necessary to preface my remarks in this way, lest it should be thought that I claim to cure anything. The most I shall attempt

* A Paper read at a Meeting of the Bristol Medico-Chirurgical Society on Wednesday, 9th January, 1935.

is to try and indicate some principles of treatment and follow up certain lines of thought which seem to me reasonable and worth considering.

Illnesses are syndromes. Perhaps some outstanding symptom is singled out in order to give a label to a condition, as for instance typhoid, locomotor ataxia, and phthisis. Actually the label matters little, provided it calls up a complete syndrome or clinical picture. We have to remember that a syndrome is a bilateral mechanism made up of two variables, invasion or trauma on the one side and reaction on the other. The reaction factor is complex and frequently baffles complete analysis. If we look at the problem of ill-health broadly we may detect reactions at various levels. At the lowest level it may be a chemical or physical one only, at the next level it is physiological, at the next psychical; we may even have to consider reactions at the highest or social level, as in the case of insanity. The important point is that our view shall be as comprehensive as possible. Reactions are adjustments, which, if successful, mean return to health. Most adjustments which concern us in illness at the physiological level are actually neuro-physiological, being mediated through an underlying mechanism for co-ordination, the nervous system. Consciousness may or may not be involved. The simplest functional unit in an organized nervous system is conduction in reflex arcs. But with advance in structural complexity of the nervous system further responses, showing higher grades of efficiency, emerge. The responses of the vegetative nervous system subserve the activities of our viscera and are essentially diffuse or widespread, ministering to instinctive cravings of the body. Response at this level is to feeling rather than to thought, so that we quite rightly appreciate emotional control exercised through the vegetative nervous system, and we must remember, therefore, that emotional control is central control, though involuntary. We cannot, for instance, determine the rate of our heart beat or our vasomotor condition apart from emotional disturbances. Many visceral adjustments acquire automatic precision; that these adjustments partake of the nature of habits appears manifest when they become deranged in functional disorders.

When we pass to the consideration of the voluntary nervous system we recognize the emergence of further capacities. The most fundamental step-up in efficiency is heralded by the appearance of the conditioned reflex. By virtue of this mechanism we become able to learn. Now, responses to significant signals become the order of the day. Skilled adjustments, memories, habits, and delayed reactions have their foundations in the conditioned reflex. Delayed reactions imply internal resistances, and without developing this line of thought in detail, one may state boldly that internal resistance is closely related to the development of consciousness. Routine activities are carried through with a minimum of conscious accompaniment, whereas more complex forms of behaviour, involving choice or decision, are apt to be associated with much internal resistance and to generate a high degree of conscious effort and emotion too. Emotional reactions overflow into the involuntary vegetative nervous system, and manifest themselves by visceral responses of a widespread character. In this way our conscious life may become inextricably mixed up with visceral responses, made manifest as physical sensations or symptoms of ill-health. The pleasure or pain in experience at the conscious level is largely determined by past pleasurable or painful associations. Of the latter fear and anxiety are the most potent. The development of fear and anxiety states is the signal for the release of widespread visceral reactions. Such reactions may become conditioned ; that is to say, on the recurrence of a situation associated by previous experience with anxiety habitual visceral disturbances are prone to appear. Now, if we remember that our viscera are vital organs, representing so to speak the internal combustion engine of the human frame, we shall appreciate how intimately our feelings of health and ill-health are connected with our emotional life. In health we are hardly conscious of having an inside at all, beyond occasional vague feelings of comfort, repletion, or physical fitness. Supposing, however, our emotional life goes wrong, our visceral field becomes the subject of bombardment by unfavourable nervous impulses, and such effects are widespread, hence the protean and diffuse character of the symptoms of the unfortunate so-called "neurasthenic." I need hardly remind you that the term "neurasthenia," is usually wrongly applied. Most of the people so labelled are the victims of continued anxiety states. They have developed unfavourable conditioned reflexes or anxiety habits. In their fear they come to expect certain dire consequences or symptoms, and they get what they fear and expect. The emotional attitudes of fear and anxiety always carry with them this element of expectation. For these people a sound aphorism would be: "Don't worry, it may never happen." It is still better if one can say: "Don't worry, it won't happen." If only they could know this for certain many of them would be cured. Often it is uncertainty that plays havoc with them. In course of time many patients lose sight of the connection between their anxieties and emotional reactions, so that ultimately physical symptoms occupy the premier position, and in this way habits of ill-health are frequently formed. If symptoms are viewed individually and treated as isolated phenomena, little benefit is likely to accrue to the patient, though the reverse may be the result in the case of the doctor.

Viewed in this way, my opening remarks become clearer. I said that the capacity on the part of a doctor to see an essential unity amidst apparent diversity is a mark of efficiency. The linkage between diverse symptoms is, of course, through the nervous system. When symptoms are multiple and diffuse in their distribution we should suspect functional derangement of the vegetative nervous system, for emotional reactions are essentially of this kind. Thus we are able to subsume a diversity of symptoms under a unifying principle, and recognize complete clinical syndromes.

I hope none of my listeners will get the impression that I am trying to whittle away the importance of organic disease. It does, however, fall to my lot to see so much of the illness-habit that I cannot avoid being on the look out for it, and, I hope, recognizing it when present. "Prejudice," you may say. I plead guilty, though I trust the tendency does not amount to an obsession. If one finds people with nothing organically amiss, and by educating them out of their fears can persuade them to take the risk of believing they are healthy, it is no bad thing.

By way of illustration of my thesis on habit and illness, I propose to consider three groups of patients as typifying certain aspects of the subject. I choose for this purpose migraine, constipation, and insomnia. That these three conditions may become habitual most of you will agree, even if you are not inclined to accept my views.

First of all, let us consider migraine. It has been the battle-ground of so much controversy and theory that it would be presumptuous to hope to dispose of it in a few minutes. The great variety of treatments recommended for migraine indicate how far we are from appreciating its full significance. Some years ago, starting from the assumption that migraine is perpetuated by habit, I decided to treat a number of consecutive cases by psychotherapy, and by psychotherapy only. As these patients did not mix their treatment, and since they were, for the most part, cases of long standing, it was possible to obtain a reasonable estimate of the value of a single form of treatment. For the immediate purpose of this paper I do not propose to discuss the original genesis of migraine, actually I believe the causes may be multiple. The point I wish to stress is, that when migraine has gone on at intervals for years it is perpetuated by habit, the various phenomena constitute a chain of conditioned reflexes if you will, which are fired off by anxiety and expectation. Each symptom in the migraine complex leads by continuous gradation into the next, the whole series of phenomena constituting the attack. On the occurrence of the initial symptom or aura the patient abandons himself to fear and expectation of the attack, and he gets what he expects as regards sequence of events, duration, etc. I have had cases of 30, 14, 10, and 7 years' duration, which had tried all the orthodox remedies without success, and have responded almost at once to psychotherapy. I have been so much impressed with the readiness with which old standing cases respond to psychotherapeutic treatment, that I now regard a history of many years as an advantage, rather than the reverse, from the point of view of favourable response to treatment. I have had less success with cases I have seen near the original attack—presumably habit has not yet crept in to any material extent in these cases. The nearer one is to the onset the more likely is one to be faced with some important physiological disturbance, possibly allergic or toxic ; but, in my opinion, such causes do not operate for years. Granted people of a certain innate nervous constitution, they react according to type, and eventually the reaction is perpetuated by pure habit with nothing else behind it. The fact that many long-standing cases respond successfully to two or three interviews, without medicine of any kind, seems to me to place the hypothesis beyond doubt.

Patients habituated to migraine come to cater for their attacks. Their detailed recital of events, and the precautions they take, all illustrate this point. Treatment consists in explaining in simple terms the nature of the nervous mechanism concerned. They must be told not to adopt their accustomed régime, but to carry on in the knowledge that the aura need not develop into a complete attack, and that if they avoid panic it will abort at the start. The majority of patients find this is actually true, and after one or two abortive turns the attacks cease entirely. This has happened too often in my experience to be a matter of chance.

I want now to pass on to the subject of habitual constipation, a problem of everyday practice. Many people, otherwise in reasonably good health, either are, or consider themselves, the subject of chronic constipation. The number of infallible laxative preparations on the market is a witness to this. Can it be really true that numbers of apparently healthy individuals need so much medicine ? I wish to exclude from the discussion certain primary organic causes of constipation, and also some secondary causes such as piles and fissure in ano, which aggravate the condition and obviously require appropriate surgical treatment. One is left with a number of cases of chronic intestinal stasis, due to a faulty neuromuscular mechanism, in which habit plays the leading rôle. The regular daily evacuation of the bowels is a habit, actually it is a conditioned reflex, established or timed-in by custom, and easily timed-out by change of routine, and notably by anxiety. The best evidence of this is Pavlov's work on conditioned reflexes. Once the habit is upset, the measures adopted in the attempt to re-establish it are frequently responsible for perpetuating symptoms. The average person attaches much importance to the state of his bowels, and rapidly becomes over-anxious when he loses his regular habit. In the early days experimentation with aperients may lead to a complete upset of the timing mechanism of the colon and rectum. Once Nature's machine is thrown out of gear patients tend to get progressively anxious and introspective. This anxiety ultimately becomes the dominant factor in perpetuating the constipation syndrome.

My interest in this subject was first aroused when working among shell-shocked soldiers after the war. I had large numbers of them under my care in Ashton Court Hospital, and among them I found that obstinate constipation was a frequent complaint. I have no doubt that in some cases, at any rate, they had lost the habit of a regular evacuation during their life in the trenches, when a visit to the latrines was apt to be postponed for obvious reasons. In any case, here were a large number of men, previously healthy, who had become obstinately constipated and were extremely worried about it. In fact, some of them seemed almost to have forgotten the war in their newly-found interest, the state of their bowels. In civil life initial personal neglect and abuse of aperients take their toll of people. One and all, sooner or later, pass into the category of anxious, miserable individuals who watch their stools with gloomy dissatisfaction. The more they watch the worse they get. At the best, the size of stool falls short of their ideal.

As regards the mechanism of the intestinal movements much has been written. The views expressed pay tribute to the ingenuity of observers and theorists. Dual nervous control through sympathetic and parasympathetic channels is freely admitted. There is, however, dispute over the details of the mechanism. Is it mainly reflex, psychical, neurohumoral, or what ?

Surgical enterprise in the treatment of chronic intestinal stasis throws considerable light on the subject. Whether by colectomy or sympathectomy, the aim of the surgeon, perhaps unconscious, is to cut out the neuromuscular control and produce a paralytic flow of faeces into the rectum, which fortunately is guarded by an external sphincter under voluntary control. The sympathectomists at least recognize that in chronic intestinal stasis the patient is defeated by faulty nervous control of the colon. They call it imbalance between the sympathetic and parasympathetic supply. I doubt, however, whether imbalance constitutes reasonable ground for removing normal nervous tissue wholesale. They seem to miss the point that the physiological stimulation of the colon is reflex and habitual. The mental accompaniments of the regularly acting colon are pleasure and confidence, whereas the mind of the constipated individual is a veritable battle-ground of anxiety (hope, fear, and uncertainty all blended), with hope at a low ebb. Sympathectomy will, I grant you, improve the situation temporarily; but the results are not lasting, a partially paralysed gut is inefficient and cannot be expected to regain normal habits. Violent purges may work, but frighten the patient. The most harmful result of chopping and changing aperients is the destruction of the habit mechanism. Few bowels can stand being whipped into activity without claiming a period of respite afterwards, and this phase of recuperation is labelled "constipation" by the patient, and calls for more aperients. Many times have I seen patients reduced to a state of distress verging on melancholia by the problem of their bowels. Careful investigation, radiological and otherwise, except for delay, may be entirely negative. A prejudice for psychotherapy led me to treat a number of these people purely on psycho-therapeutic lines. Results have seemed to justify this line of treatment.

It is important to spend some time teaching them the physiology of the normal mechanism and by simple psychotherapeutic measures persuade them that their condition is not serious, but the result of loss of habit. Let them realize that they still possess a perfectly normal apparatus capable of being restored to proper functional efficiency, if only they would give it a chance.

Of course, patients like to feel that there is something active left for them to do in the matter. Accordingly, they are instructed to resort to a few simple routine measures. (1) They should drink cold water freely on rising in the morning and carry out a series of physical exercises when dressing. This serves to initiate normal gastrocolic reflex. (2) They are taught to retire to stool at a definite time each day and allow a sufficient time to be taken over the process. They are forbidden to retire at frequent irregular intervals during the day in the hope that something may happen, for such conduct is pandering to anxiety or panic. (3) They are forbidden to take aperients, though, at the beginning of treatment, they are allowed a glycerine suppository to initiate the reflex at a fixed time of day.

Let me pass on to the consideration of chronic insomnia, a condition which frequently crops up in our daily practice. One must be careful to exclude from the discussion sleeplessness occurring as a phase of acute illness, such as pneumonia or acute abdominal disease, or associated with severe physical pain, more particularly, perhaps, pain in the head. There is, too, sleeplessness, a prodromal symptom of mania and other major mental disorders. Excluding these, there remains a large number of patients who sleep badly, so badly indeed that they become distraught or, at least, very anxious about it. The causes of chronic insomnia among the last class of patients are anxiety and bad habit. The abuse of hypnotics figures largely in the problem.

One may with advantage study the psychology of these people. The starting-point is usually some very genuine anxiety. Reviewing some of my cases, I find under this heading : (i) disturbed nights, owing to the prolonged nursing of some sick relation ; (ii) family bereavement, *e.g.* the loss of a child ; (iii) business worries ; (iv) moral conflict ; (v) war memories. These last are now rare, but are represented by the type of insomnia associated with nightmares. It is a matter of common experience that continued anxieties, which are not properly faced, owing to indecision, regret or remorse, and so forth, lead to sleeplessness. When the patient lacks courage or decision there is a tendency for the anxiety problem to be repressed into the background or fringe of consciousness during the daytime, and by night this unfavourable emotional material wells up into consciousness and prevents sleep. It is fairly obvious that sedatives and hypnotics cannot settle anxiety problems, and that they can hardly be expected to cure the insomnia of a patient whose mind is in a turmoil. The matter is, however, not quite so simple as this. The continued use of hypnotics leads eventually to tolerance, so that in time each drug in turn loses its effect. The patient becomes more and more distracted by the failure of drugs to produce sleep, apart from their total failure to restore the habit of sleep. In this way a secondary system of anxiety is induced. For this class of patient sleep is the one thing he feels necessary to mental salvation. He argues : " If I don't sleep I shall have a complete nervous breakdown, and perhaps become insane," thus in time he panics about not sleeping. Alternatively, he fears that he may become a drug addict. Patients with war memories, or kindred distressing experiences in civil life, are liable to be disturbed by terrifying nightmares. On account of the latter, they may be afraid of going to sleep at all. They find themselves faced with a dilemma. If they sleep they will wake up in terror, and if they don't sleep they will go off their heads.

Before passing on to treatment, let us consider quite briefly the mechanism of normal sleep. In the ordinary way we sleep from established habit. Sleep is a conditioned inhibition of our highest cortical centres. Monotony of our surroundings plays an important part in the induction of such inhibition. The accustomed bed and surroundings are a help. Noise, though often blamed, is seldom responsible for sleeplessness, for customary noise, such as the roar of traffic and proximity to a railway, soon ceases to claim our attention, though a significant slight noise may do so at once. A mother will sleep through a medley of street noises, but if her child stirs in the cot by her side she is wide awake at once. It is wrong to think that fatigue and exhaustion help in the production of normal sleep. Most of us sleep because we go to bed whether we are tired or not. Brain workers as a whole sleep more lightly and for shorter hours than manual workers. This is, perhaps, contrary to expectation.

The behaviour of victims of chronic insomnia is interesting, and in the main exhibits much uniformity. At any rate, the devices they adopt to secure sleep are limited in number. Here are some of them: the single room, the hot drink on retiring, the extra warm bed, thick curtains, and cotton wool in the ears to exclude noises, reading in bed, counting, etc., etc. Many a patient will say: "I go to bed determined to sleep and make every effort to relax." It is, perhaps, well to remind them:—

> "Sleep is no servant of the will,
> It has caprices of its own;
> When least desired it lingers still,
> When most pursued 'tis quickly gone."

Now as regards treatment: the most important aim is to allay anxiety. How can this be done? Detailed and truthful explanation is all important. I tell them that people don't break down from chronic sleeplessness, because Nature always ensures the necessary minimum of sleep; if they tried to keep awake they would fail, because absolute sleeplessness is physiologically impossible. Let me remind you again that acute insomnia is excluded from the discussion. All patients who sleep badly are poor witnesses as to the amount they sleep, they all err in the direction of underestimating it. Secondly, I advise all patients with disturbing problems to face them courageously during their waking hours instead of repressing them. The latter proceeding is like putting one's unpaid bills in the waste-paper basket, they are sure to come in "account rendered." The next thing is to point out that the very measures they adopt to secure sleep are a tribute to fear, such measures have already proved a failure, and the mere act of carrying them out is a powerful auto-suggestion that they are not going to sleep. Cotton wool or wax placed in the ears is a particularly pernicious habit. Not only does it fail to exclude noise, but actually it induces hyperacusis. The patient unwillingly listens in order to detect what sounds he can hear through the obstruction. The proceeding finds its parallel in the neurasthenic, who adopts smoked glasses. His eyes become eventually so sensitive that they are dazzled by light of even low intensity. Again, effort at relaxation is the wrong way to set about it, absence of effort is the attitude necessary for relaxation. The patient must be taught to cultivate a complete indifference as to whether he sleeps or not.

The question may be asked: "Have drugs any place in the treatment of habitual insomnia?" Speaking generally, I think the correct answer is "No." In practically all chronic cases drugs have already failed and are partly responsible for the demoralization of the patient. By the mere act of prescribing you undermine the patient's confidence in your psychotherapeutic dicta. To summarize the principles of treatment. In the first place try and deal satisfactorily with the patient's attitude towards important primary anxieties. Make him realize he has lost a habit and no more. Teach him a healthy indifference towards the sleep problem, emphasize that nature always ensures the necessary minimum of sleep. What he gets beyond this is a luxury, so to speak, and he can well afford to be indifferent about it. This healthy indifference is just the attitude calculated to induce mental and physical relaxation which merges into the state of cortical inhibition we call sleep.

I appreciate that this apparently negative line of treatment may meet with criticism. At the onset of sleeplessness it is often simple and satisfactory to prescribe sedatives and hypnotics and the patient gets well. I quite agree, but there will be a considerable residue of cases which do not prove so easily amenable. The sleep habit once seriously broken is not readily reformed, and drugs won't do it. Each year that passes I see a number of such cases and from a somewhat prolonged experience I am convinced that relief must be found by the re-education of the patient in normal habit through psychotherapy.

In conclusion, in taking as my text "Habit and Illness" I have made it the opportunity to bring to your notice certain aspects of continued ill-health, which find a reasonable explanation in faulty habit formation. It falls to my lot to see many cases of the kind I have mentioned. For the most part these patients have run through the gamut of pharmacopœial treatment and they stand out as failures. For this reason I have chosen to treat them by psychotherapy only. A considerable measure of success has led me to conclude that the hypothesis that they are suffering from faulty habit and faulty habit only seems justified. In stressing the usefulness of psychotherapy I am not suggesting that drugs and a general medical régime play no part, but that whatever treatment is adopted it must rest on the foundation of re-education of the patient's outlook. To treat individual symptoms is useless. Habits are conditioned reflexes, that is to say, responses to significant signals, formed by past associations. They may involve conscious or unconscious levels of the nervous system. When associations are markedly tinged with anxiety, diffuse reactions occur in the field of vegetative nervous system, producing symptoms of physical ill-health. Frequently, it is these physical symptoms which bring the patient to the doctor, and the associated anxiety state is only elicited on inquiry. The only way to stop such reactions is to educate the patient into a clear understanding of their significance. By this means anxiety is allayed and the path is open for recovery. It is obvious that some degree of intelligent co-operation on the part of the patient is essential to success. One must exercise discrimination in choosing patients if waste of time is to be avoided. Perhaps the chief impediment to successful psychotherapy is advanced years. Habits good and bad become deeply ingrained by fifty ; but more important than this is the fixed mental outlook of the elderly patient, he is seldom capable of readjusting his ideas, and is, therefore, a bad subject for re-education.

